# Single channel properties of mitochondrial large conductance potassium channel formed by BK-VEDEC splice variant

**DOI:** 10.1038/s41598-021-90465-3

**Published:** 2021-05-25

**Authors:** Shur Gałecka, Bogusz Kulawiak, Piotr Bednarczyk, Harpreet Singh, Adam Szewczyk

**Affiliations:** 1 Laboratory of Intracellular Ion Channels, Nencki Institute of Experimental Biology, Polish Academy of Sciences, 3 Pasteur St., 02-093 Warsaw, Poland; 2 Department of Physics and Biophysics, Institute of Biology, Warsaw, University of Life Sciences-SGGW, Nowoursynowska 166, 02-787 Warsaw, Poland; 3 Department of Physiology and Cell Biology, The Ohio State University, Columbus, OH 43210 USA

**Keywords:** Biochemistry, Biophysics

## Abstract

The activation of mitochondrial large conductance calcium-activated potassium (mitoBK_Ca_) channels increases cell survival during ischemia/reperfusion injury of cardiac cells. The basic biophysical and pharmacological properties of mitoBK_Ca_ correspond to the properties of the BK_Ca_ channels from the plasma membrane. It has been suggested that the VEDEC splice variant of the *KCNMA1* gene product encoding plasma membrane BK_Ca_ is targeted toward mitochondria. However there has been no direct evidence that this protein forms a functional channel in mitochondria. In our study, we used HEK293T cells to express the VEDEC splice variant and observed channel activity in mitochondria using the mitoplast patch-clamp technique. For the first time, we found that transient expression with the VEDEC isoform resulted in channel activity with the conductance of 290 ± 3 pS. The channel was voltage-dependent and activated by calcium ions. Moreover, the activity of the channel was stimulated by the potassium channel opener NS11021 and inhibited by hemin and paxilline, which are known BK_Ca_ channel blockers. Immunofluorescence experiments confirmed the partial colocalization of the channel within the mitochondria. From these results, we conclude that the VEDEC isoform of the BK_Ca_ channel forms a functional channel in the inner mitochondrial membrane. Additionally, our data show that HEK293T cells are a promising experimental model for expression and electrophysiological studies of mitochondrial potassium channels.

## Introduction

Mitochondrial potassium (mitoK) channels play an important role in the physiology of mitochondria. The activation of mitoK channels results in K^+^ influx into the mitochondrial matrix which decreases the mitochondrial membrane potential and can thus affect various processes such as oxidative phosphorylation and reactive oxygen species synthesis^1,2^.

Several potassium channels have been identified in the inner mitochondrial membrane^1,3^. The basic biophysical and pharmacological properties of mitoK channels are very similar to those of their plasma membrane counterparts. One of the best-described channels in mitochondria is the large conductance calcium-activated potassium (mitoBK_Ca_) channel. For the first time mitoBK_Ca_ was identified by the patch-clamp technique in the mitochondria of glioma cells^4^. Later, the channel was described in other tissues, including the heart, brain, endothelium, skin fibroblasts or skeletal muscle and in the mitochondria of lower organisms^1,5–11^.

The mitoBK_Ca_ channel plays a cytoprotective role that is most apparent during hypoxia/reperfusion injury of heart tissue^11–15^. The detailed mechanism of this phenomenon is unclear; however it was suggested that the mild uncoupling of the mitochondrial membrane potential induced by mitoBK_Ca_ opening might influence mitochondrial reactive oxygen species synthesis and/or prevent mitochondrial calcium overload^11,13,15–18^. It was also observed that mitoBK_Ca_ might be the target for multiple signaling pathways including phosphorylation and redox regulation, which might be critical for the induction of cytoprotective mechanisms^15,19,20^.

The biophysical properties of mitoBK_Ca_ expressed natively were described using electrophysiological tools including mitoplast patch clamping and reconstitution of submitochondrial particles into bulk lipid membranes^21–23^. Usually the mitoBK_Ca_ conductance is close to 300 pS however in heart tissue, it was also shown to be below 200 pS^12^. The open probability of the channel depends on the voltage and concentration of calcium ions. The channel is activated by several potassium channel openers such as NS1619, NS110021 or CGS7184 and inhibited by iberiotoxin, charybdotoxin and paxilline^24–26^. Similar to the plasma membrane BK_Ca_ channels, mitoBK_Ca_ channel activity is reduced by heme and hemin^27–29^. All of the above properties correspond to the biophysical and pharmacological properties of BK_Ca_ channels in the plasma membrane^30,31^.

The BK_Ca_ channels in the plasma membrane are tetramers formed by pore-forming α subunits encoded by the *KCNMA1* gene. The properties of the channels can be modified by β and γ auxiliary subunits^31,32^. Based on similarities between mitoBK_Ca_ and BK_Ca_ in the plasma membrane, it was proposed that the α subunit of both channels is encoded by the same gene. These observations were supported by identification of the α protein in the mitochondrial fraction with antibodies recognizing the subunit of the plasma membrane BK_Ca_^5,6,9,33^. However, the *KCNMA1* gene was reported to produce multiple splice variants. Several recent studies proposed that one of the mitochondrial isoforms of the α subunit might be a protein containing the VEDEC tail motif. This isoform was shown to colocalize with mitochondria in the mouse cochlea^34,35^ and rat heart^36^. Similarly, expression of this isoform in HEK293T cells resulted in partial localization of this protein to the mitochondria^37^; however, there was no direct evidence that this splice variant forms a functional channel in mitochondrial membranes. Here, we provide a set of experimental data describing the channel activity formed by the VEDEC isoform in the mitochondrial inner membrane. In our study, we used HEK293T cells transiently transfected with a plasmid encoding this splice variant^36,37^ followed by a patch-clamp of isolated mitochondria. Electrophysiological recordings of detected activity correspond with known properties of the mitoBK_Ca_ described previously. Therefore, we conclude that the VEDEC splice variant is able to form the mitoBK_Ca_ channel in the inner mitochondrial membrane. Additionally, our data indicate that HEK293T cells are a very useful experimental model for the expression and electrophysiological recordings of mitochondrial potassium channels.

## Results

The main aim of this study was to measure activity and to characterize BK_Ca_ channel previously described as mitochondrial isoform of BK_Ca_ channel, namely, VEDEC channel.

To achieve the intended goal, we used the following approach:

- expression of the VEDEC BK_Ca_ isoform in cells lacking any background K^+^ channel activity in the inner mitochondrial membrane;
- confirming mitochondrial localization of the a VEDEC isoform;
- describing basic BK_Ca_ channel properties such as regulation by known channel activators and inhibitors.

### Biophysical properties of mitochondrial channels recorded after transfection with VEDEC-encoding plasmid

**Figure 1:**
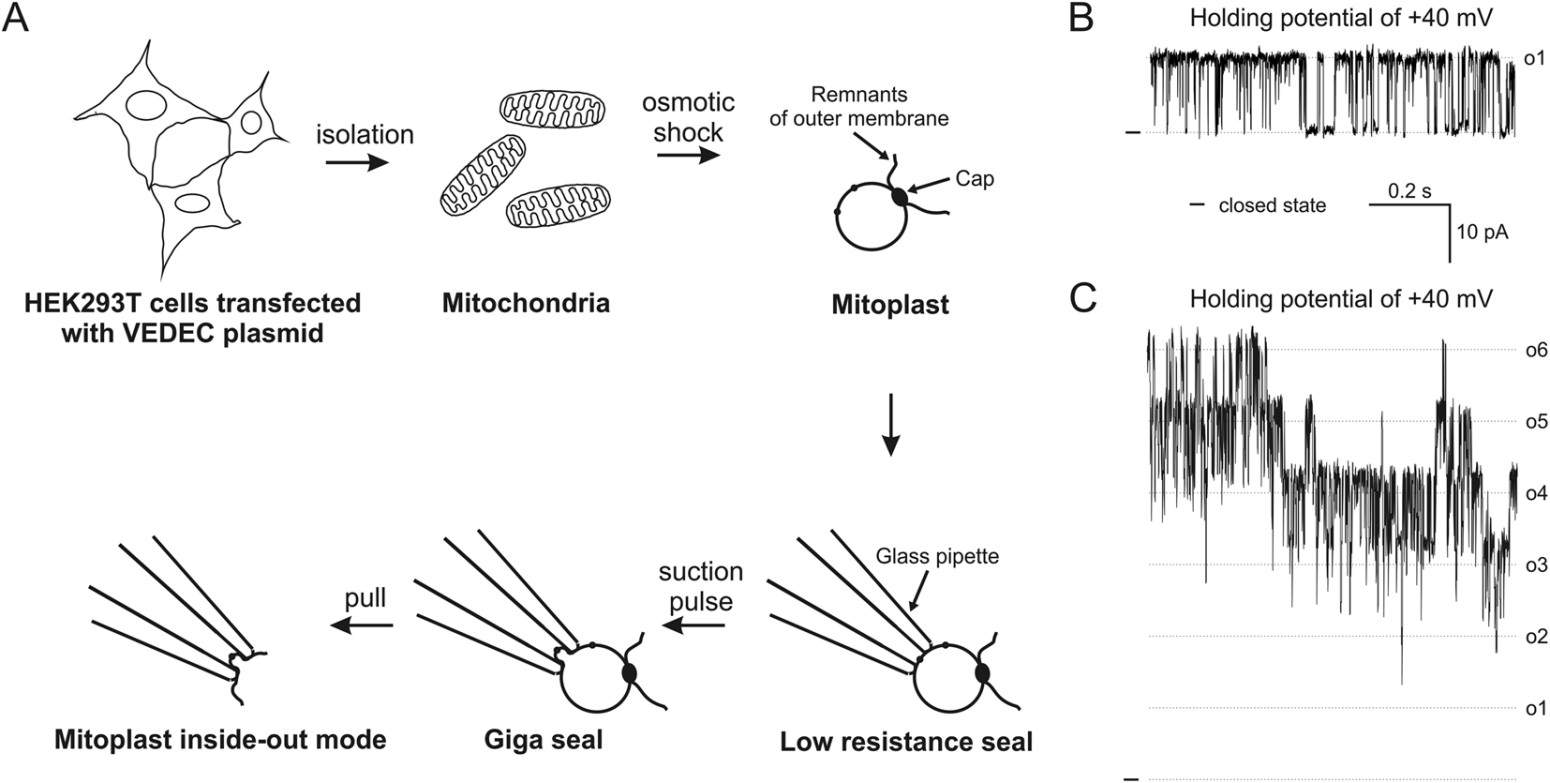
Mitoplast patch-clamp measurement and single-channel recordings of channels observed after transient transfection with the BK_Ca_ VEDEC-encoding plasmid. (**A**) Schematic depiction of the patch-clamp channel measurement of the inner mitochondrial membrane: mitoplast preparation and single-channel patches in the inside-out configuration. (**B**) Representative recording of single- and (**C**) multiple-channel activity in the patch of the inner mitochondrial membranes after transient transfection of HEK293T cells with the BK_Ca_ VEDEC-encoding plasmid. Single- and six-mitoBK_Ca_ channel activities are presented. Recordings were performed at holding potential + 40 mV.

In the present study, we used HEK293T cells as a model system for the expression of the VEDEC isoform of the protein encoded by the *KCNMA1* gene. This cell line is a known model system for plasma membrane BK_Ca_ research. To verify the hypothesis that HEK293T cells could be an appropriate experimental model for mitoK channel studies, we decided to perform a series of experiments using mitoplast patch-clamp measurements. In our studies, we used mitochondria isolated from HEK293T cells transiently transfected with a construct encoding the VEDEC isoform of BK_Ca_ described previously^36,37^. A schematic depiction of the experimental protocol used in the study is presented in Fig. 1A. The ion current was measured in symmetrical 150/150 mM KCl isotonic solution in the presence of 100 µM Ca^2+^ unless otherwise stated. We found that transient transfection of HEK293T cells with a plasmid encoding VEDEC resulted in recordings of single or multiple channels with kinetics shown in the Fig. 1B and C. By contrast, we did not detect similar ion channel activity in mitochondria isolated from untransfected HEK293T cells (n = 104 patches, 10 mitochondrial isolations).

**Figure 2:**
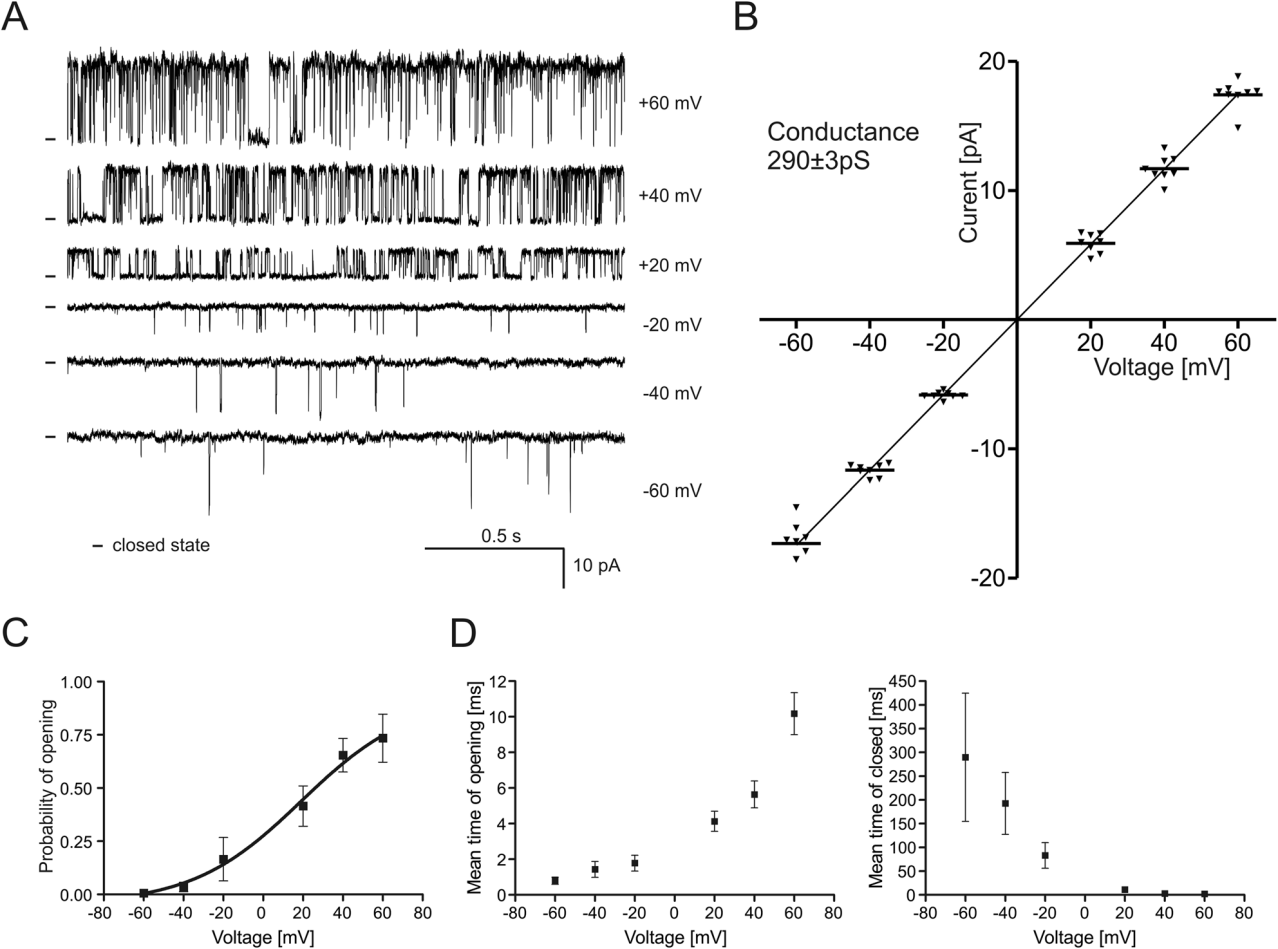
Biophysical properties of the channel recorded in mitoplasts isolated from HEK293T cells transiently transfected with the VEDEC isoform of the BK_Ca_ channel. (**A**) Single-channel recordings in symmetrical 150/150 mM KCl at different voltages ranging from -60 to + 60 mV. (**B**) Current–voltage characteristics of the single-channel events in symmetrical 150/150 mM KCl. The conductance of the channel, as calculated based on the presented I-V curve, was equal to 290 ± 3 pS. (**C**) Analysis of the channel open probability (P_o_) at different potentials. (**D**) Distribution of the mean open and closed dwell times of the channel at different voltages in symmetrical 150/150 mM KCl solution. The results are presented as the means ± SD (n = 7).

The calculated conductance of the channel based on the current/voltage dependence (Fig. 2A,B) was equal to 290 ± 3 pS (n = 7). Interestingly, recordings indicating the activity of multiple channels (up to approximately 30 channels in the single patch) were frequently detected. In the first set of experiments we analyzed the dependence of the channel activity on the applied voltage (Fig. 2C). The opening probability of (P_o_) of the channel was approximately 0.006 at − 60 mV and a gradual increase in P_o_ was observed when a more positive voltage was applied. The maximal P_o_ was observed at + 60 mV and was equal to 0.73. Experimental points presented in the Fig. 2C were fitted using Boltzmann function. Based on fitting results, calculated value of potential at which open probability is halfway (V_1/2_) was 19.2 mV and gating charge was equal to 0.95e_0_.

Application of wider range of voltages resulted in patch instability and disruption of the membrane. Additionally, the mean open time and mean closed time dwell times at various voltages were calculated (Fig. 2D). The maximal average τ_open_ was observed at + 60 mV and was equal to 10.2 ms, and the maximal average τ_closed_ was observed at − 60 mV and was equal to approximately 289.7 ms. These biophysical properties of the channel were conducted base on single-channel recordings (n = 7).

Based on the above data we concluded that the biophysical properties of the mitochondrial ion channel recorded after transfection with the VEDEC encoding plasmid correspond with the properties of the previously described mitoBK_Ca_ channel in various tissues^1^.

### The channel formed by VEDEC in mitochondria is Ca^2+^-regulated

**Figure 3:**
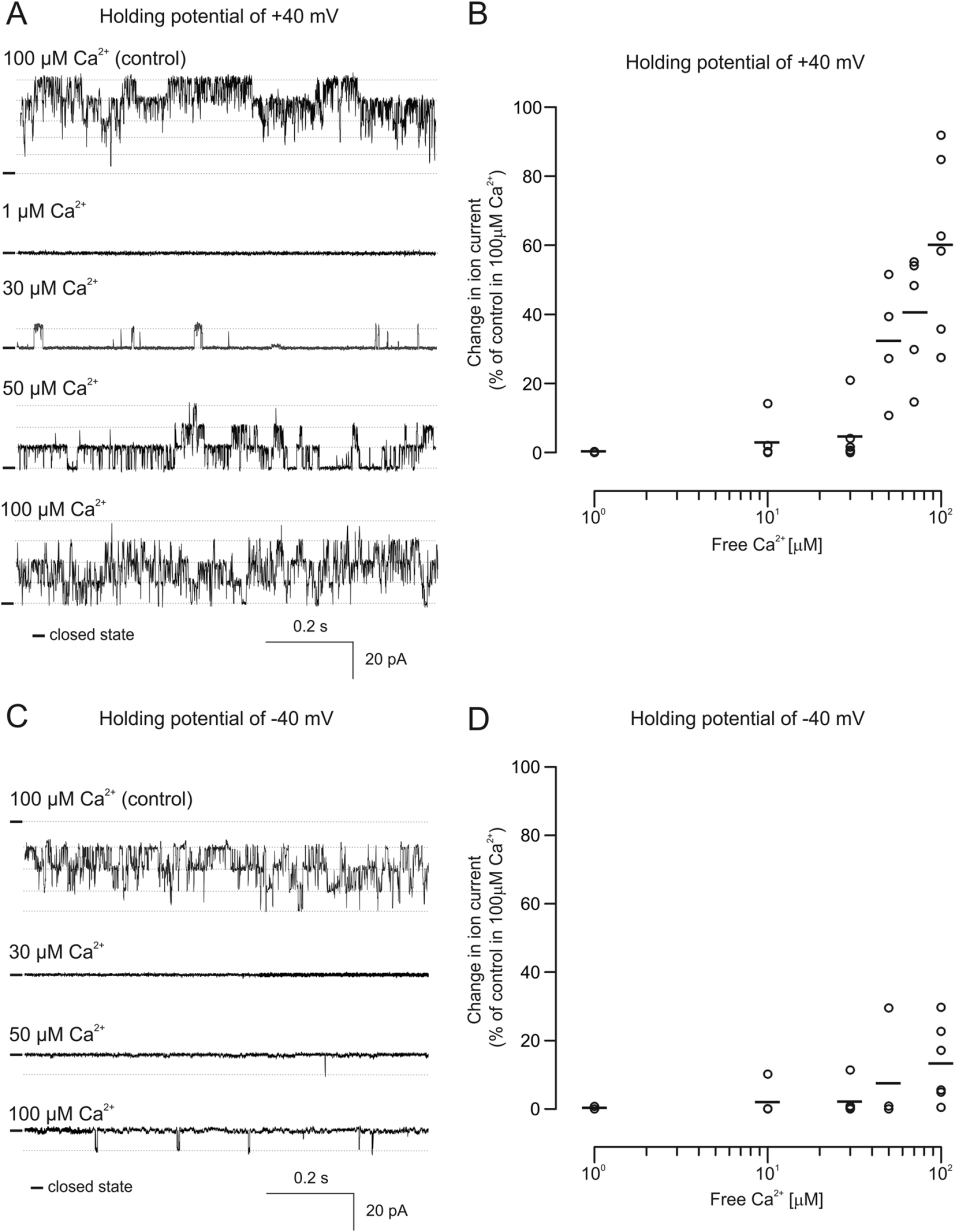
Modulation of the channel activity by calcium ions. (**A**) Sample of the single-channel recordings in symmetrical 150/150 mM KCl solution at different Ca^2+^ concentrations at a holding potential of + 40 mV. (**B**) Analysis of changes in the ion current in the presence of various Ca^2+^ concentrations recorded at holding potentials of + 40 mV. (**C**) Representative single-channel recordings in symmetrical 150/150 mM KCl solution in the presence of different Ca^2+^ concentrations recorded at a holding potential of − 40 mV. (**D**) Analysis of changes in the ion current in the presence of various Ca^2+^ concentrations recorded at a holding potentials of − 40 mV.

In the second set of experiments, we verified the dependence of the channel on the presence of calcium ions, which is the canonical property of mitoBK_Ca_ channels. For this purpose, the channel activity was recorded in a buffer containing various concentrations of free calcium ions (Fig. 3). The activity of the channels was recorded at holding potentials equal to + 40 mV or − 40 mV. Perfusion of the channel with buffer containing 1 µM free Ca^2+^ resulted in a virtual lack of channel activity (0.07% of control at + 40 mV and 0.28% of control at − 40 mV). Increasing the free Ca^2+^ in the buffer to 50 µM resulted in an ion current boost to approximately 32.2% of that of the control at + 40 mV and 7.6% at − 40 mV. When the level of free Ca^2+^ returned to the control level (100 μM) observed ion current increased up to 60.2% of that of the control at + 40 mV and only 13.5% at − 40 mV. We also noticed large variability in channel activity in response to increasing calcium ion concentrations in the applied buffer. Nevertheless our data clearly show that the channel recorded after transient transfection with the VEDEC plasmid shows strong Ca^2+^ regulation.

### Regulation of the channel formed by the VEDEC splice variant by mitoBK_Ca_ modulators

**Figure 4:**
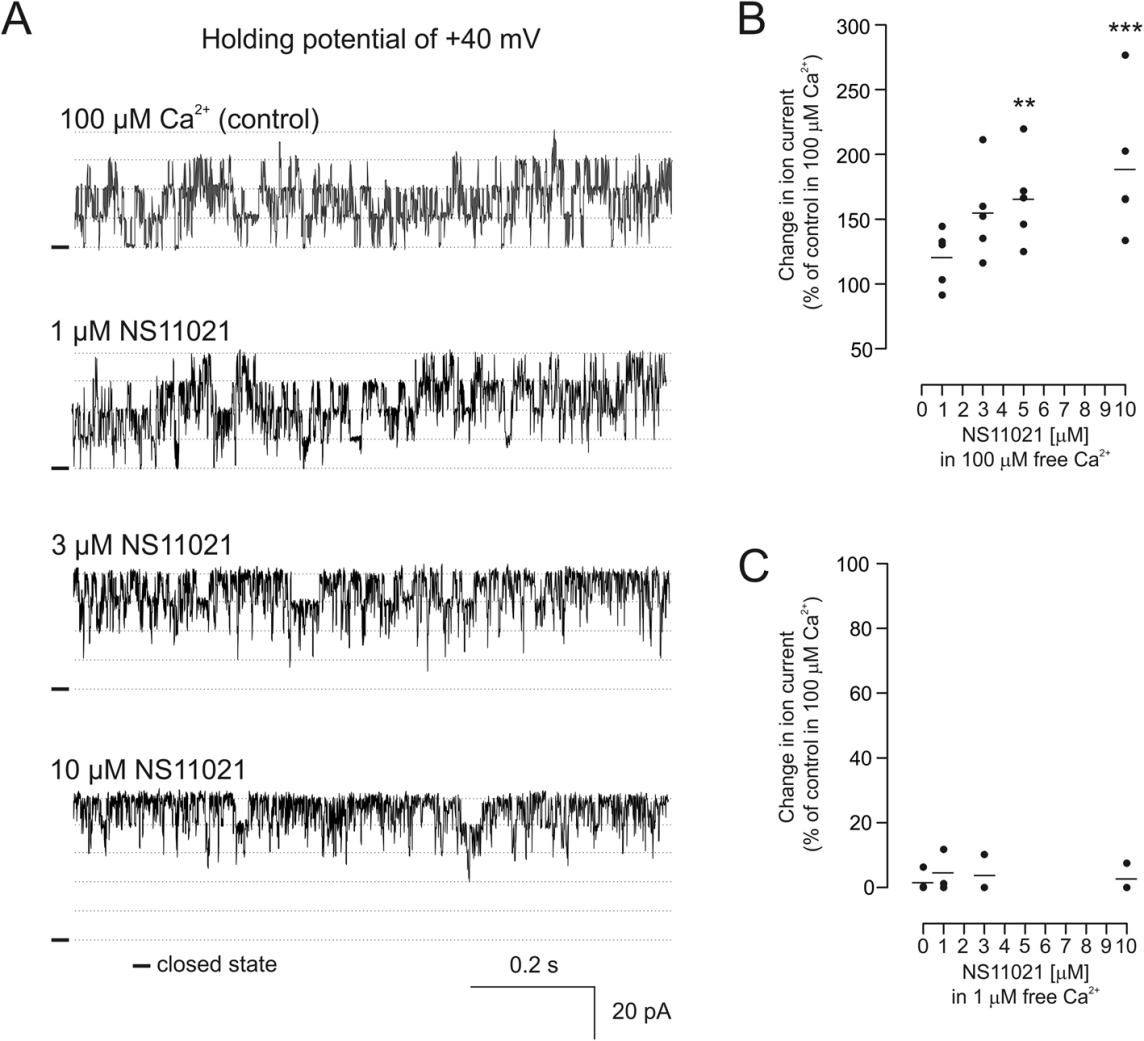
Regulation of channel activity by the BK_Ca_ channel activator NS11021. (**A**) Single-channel recordings in symmetrical 150/150 mM KCl solution at different NS11021 concentrations in the presence of 100 µM Ca^2+^ in experimental buffer at a holding potential of + 40 mV. (**B**) Analysis of changes in the ion current induced by increasing NS11021 concentrations in the presence of 100 µM Ca^2+^ recorded at holding potential of + 40 mV. (**C**) Analysis of changes in the ion current induced by increasing concentrations of NS11021 recorded in the presence of 1 μM Ca^2+^. **p < 0.01, ***p < 0.001 vs control activity in 100 µM Ca^2+^.

In the next step, we characterized the pharmacological properties of the channel formed by the VEDEC isoform. Therefore, typical modulators of the mitoBK_Ca_ channel were applied. In our research, we used NS11021, which was shown to activate channels both from the plasma membrane and from mitochondria^7,38,39^. The first set of experiments was performed in the presence of 100 µM calcium ions in the buffer. The application of NS11021 induced channel activation in a concentration-dependent manner (Fig. 4A). A maximal increase in the ion current was observed after the application of 10 µM NS11021 at + 40 mV (approximately 175% of the control) (Fig. 4B). Interestingly, when the free calcium ion concentration was reduced from 100 µM to 1 µM, the application of NS11021 resulted in very modest activation of the channel (Fig. 4C).

**Figure 5:**
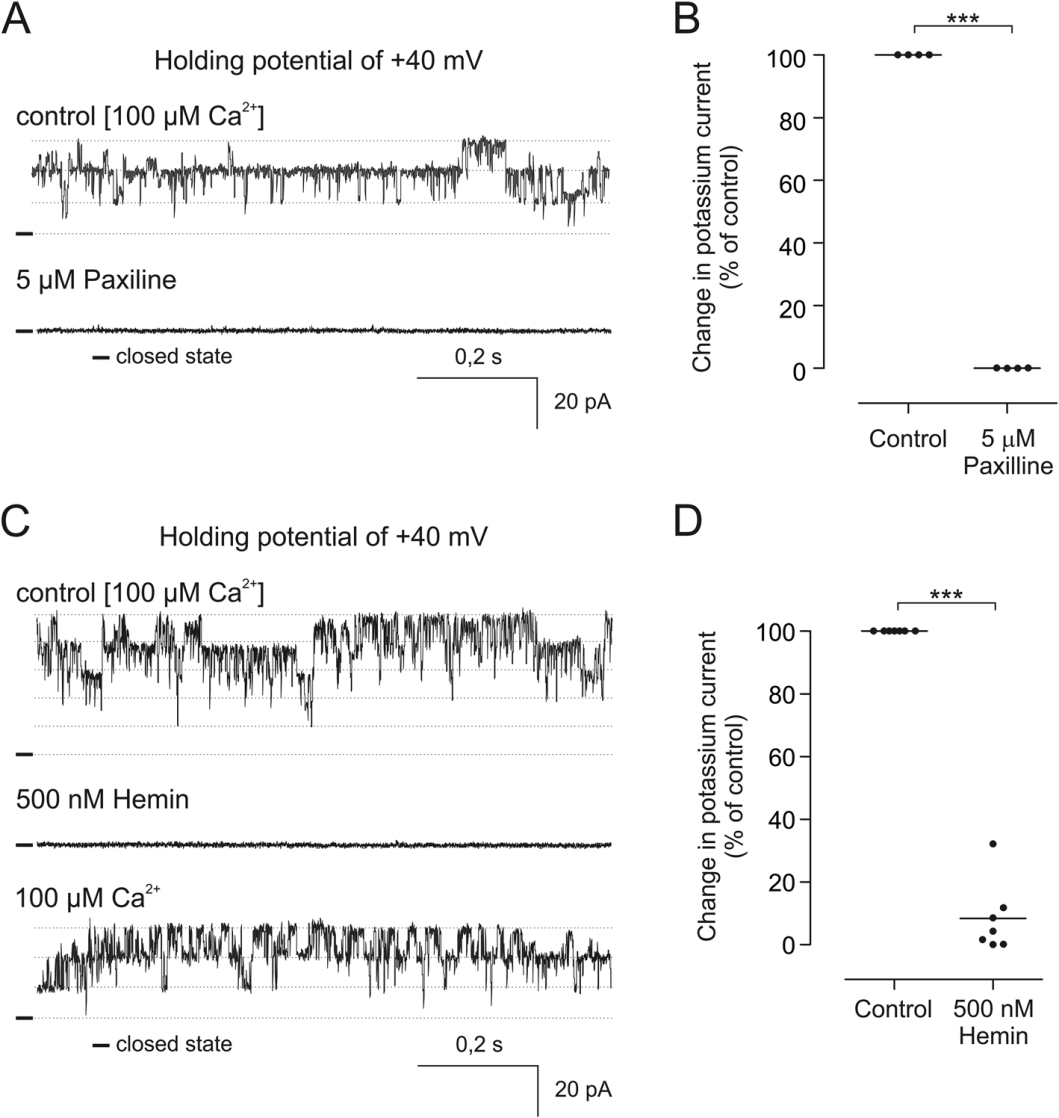
Inhibitory effect of the BK_Ca_ channel blockers on the channel recorded after transfection with VEDEC. (**A**) Single channel recording in symmetrical 150/150 mM KCl of the channel under the control condition and after 5 µM paxilline application. (**B**) Statistical analysis of the paxilline inhibitory effect on the recorded channel. (**C**) Representative single- channel recording of the channel in symmetrical 150/150 mM KCl under the control condition and after 500 nM hemin application (**D**) Analysis of changes in the ion current under the control condition and after hemin application. ***p < 0.001 vs control activity.

Contrary to the potassium channel opener NS11021, the fungal tremorgenic alkaloid paxilline is the canonical inhibitor of BK_Ca_ channels^23,40–42^. Therefore, we applied this blocker to the channel recorded in our experimental system. All recorded channels with pharmacological and biophysical properties corresponding to mitoBK_Ca_ were blocked by 5 µM paxilline (Fig. 5).

Furthermore, it has been shown that BK_Ca_ channels contain a heme-binding motif in their C-terminal part, and the application of hemin results in the inhibition of these channels, including mitoBK_Ca_^27,43^. Our experiments revealed that the application of hemin indeed resulted in clear inhibition of the channels recorded after transfection with the VEDEC isoform. When the channel was recorded at + 40 mV in the presence of 100 µM free calcium ions, perfusion of the patch with 500 nM hemin resulted in a decrease in the ion current to approximately 8,3% of the control value (Fig. 5A,B).

### Analysis of the BK_Ca_ expression in HEK293T cells

**Figure 6:**
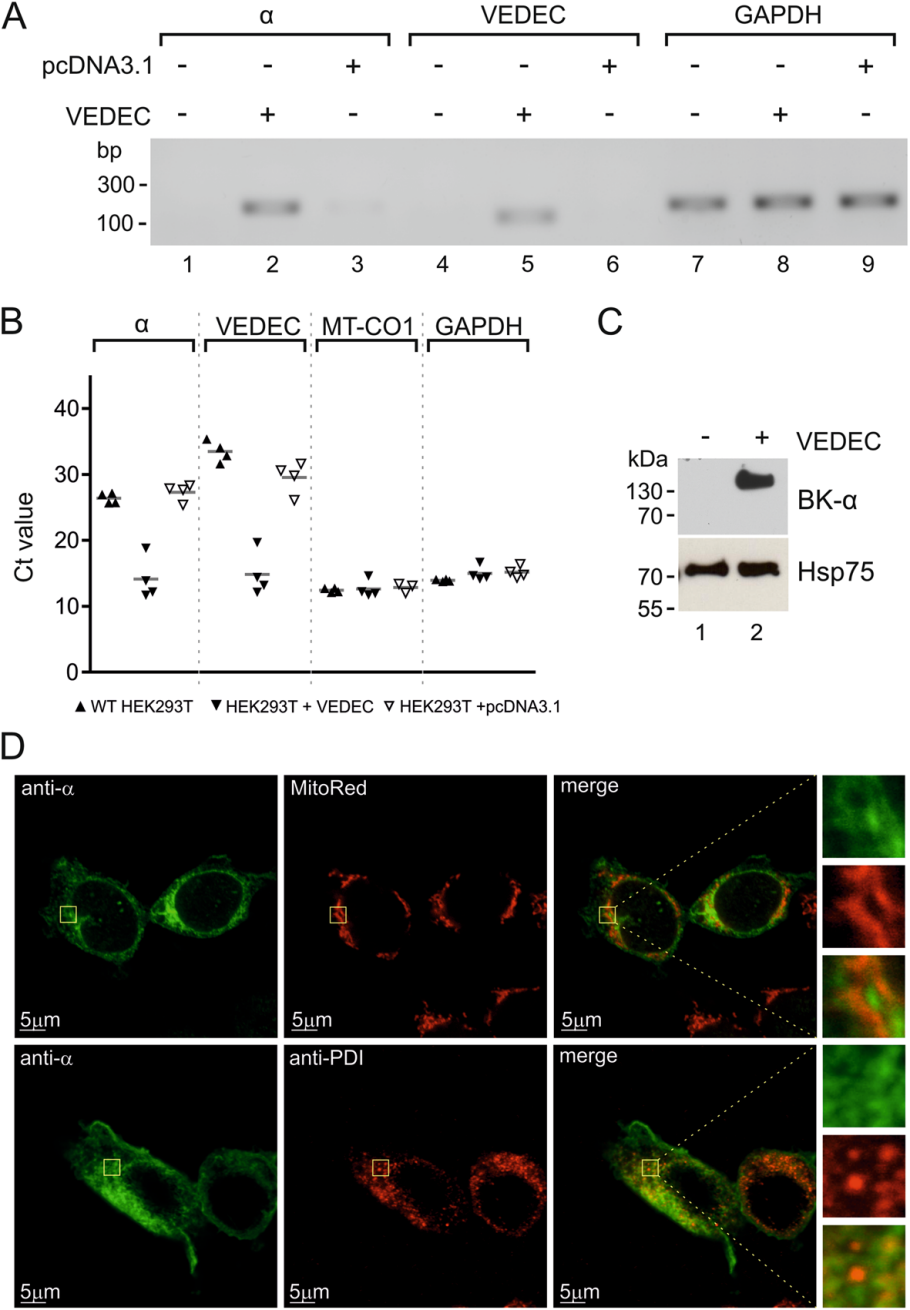
Analysis of the presence of the BK_Ca_ α subunit in wild-type and VEDEC transiently transfected HEK293T cells. (**A**) Analysis of selected gene expression using qualitative PCR. (**B**) Real-time PCR analysis of the expression levels of selected genes. (**C**) Western blot analysis of the BK_Ca_ pore-forming subunit in the mitochondrial fraction of wild-type and transfected cells. (**D**) Localization of VEDEC in HEK293T cells after transient transfection. Confocal images of cultured cells stained with MitoRed as a mitochondrial marker (upper panel, red channel) and an anti-PDI antibody as an endoplasmic reticulum marker (lower panel, red channel). The pore-forming α subunit was stained with an anti-BK α antibody (green channel). The superimposition of the two signals revealed the partial mitochondrial and ER localization of the BK_Ca_ α subunit (orange).

To verify our electrophysiological mitoplast patch-clamp experiments we decided to analyze the expression of pore-forming BK_Ca_ channel subunits and, specifically, VEDEC isoform in wild-type and transiently transfected HEK293T cells (Fig. 6A,B). Previous reports suggested either that the expression of the BK_Ca_ channel pore-forming subunit in wild-type HEK293 cells is not detectable^40^ or that the BK_Ca_ currents were at very low levels in comparison to transfected cells^41^. However, recently, it was also reported that natively expressed BK_Ca_ might be present in the mitochondrial fraction of HEK293 cells^44^. In our study we used HEK293T cells which are a modified subclone of the original HEK293 cell line^45^. In our experiments, we used primers recognizing all splice variants of the pore-forming subunit (marked as “α”) and VEDEC isoform (marked as “VEDEC”). Standard qualitative PCR experiments suggested that in wild-type cells, the expression of the *KCNMA1* gene product was virtually undetectable, whereas transient transfection of HEK293T cells resulted in high expression of the BK_Ca_ α subunit (Fig. 6A, S1). However, quantitative PCR suggested endogenous expression of the α component in wild-type cells (Fig. 6B). This result was obtained using both sets of primers. Transfection with VEDEC resulted in multiple increases in the expression of the BK_Ca_ α subunit (shift in Ct value). For comparison, the expression of control genes (GAPDH and MT-CO1) did not change significantly under all tested conditions. Due to ambiguous quantitative PCR results in wild-type HEK293T cells, we decided to perform western blot analysis of α protein levels. In the nontransfected cells, we were unable to detect the α subunit of the BK_Ca_ channel. On the other hand, transfection of HEK293T cells with the VEDEC splice variant resulted in the presence of the BK_Ca_ channel pore-forming subunit in the crude mitochondrial fraction (Fig. 6C, S2).

### Subcellular localization of VEDEC in HEK293T cells

In the last set of experiments we wanted to verify the mitochondrial localization of BK_Ca_ α expressed in transiently transfected HEK293T cells. Immunostaining followed by confocal microscopy revealed that mitochondria are one of the targets of the VEDEC protein (Fig. 6D). Additionally, we observed colocalization of the α subunit with the endoplasmic reticulum. In our opinion, immunostaining experiments confirmed that the expression product of the VEDEC plasmid is partially targeted toward mitochondria.

## Discussion

Despite many years of research, many questions remain concerning the structure and function of the mitoBK_Ca_ channel^3^. It has been shown that the VEDEC isoform of the BK_Ca_ channel is located in the mitochondria of heart tissue^36^. However, there was no evidence that this protein could form an active channel in the inner mitochondrial membrane. The research presented in this paper aimed to verify this hypothesis.

In our study, we used a mitoplast patch-clamp to detect ion channel activity resulting from transfection of HEK293T cells with a plasmid encoding a VEDEC isoform of the BK_Ca_ channel.

Based on the data obtained, two main conclusions can be drawn. First, the expression of the VEDEC isoform leads to mitoBK_Ca_ activity in the mitochondrial inner membrane. Second, our data show that HEK293T cells are potentially a suitable model for expression and electrophysiological studies of mitoK channels.

The channel observed after transfection with VEDEC showed a conductance of approximately 290 pS which is close to the conductance previously described for the BK_Ca_ channel. In brain tissue and several glioma cell lines the average conductance of the channel recorded under the same experimental conditions ranged between ~ 275 pS and ~ 295 pS^4,8,23,46–48^. Similar values were reported for mitoBK_Ca_ from skeletal muscle and endothelial cells^6,7^. The conductance of the recorded channel was also close to the values observed in the plasma membrane in stably transfected HEK293 cells where a 250pS channel was reported after the expression of BK_Ca_^49^. However, the conductance of mitoBK_Ca_ can differ from that indicated above. For example, in mouse heart tissue, conductance of the channel was significantly smaller and did not exceed 190 pS depending on the experimental conditions^12,19^. On the other hand, a conductance of 308 pS was reported for the mitoBK_Ca_ channel from rat heart cardiomyocytes^22^.

Other analyzed biophysical features of the recorded channels are also similar to those reported for mitoBK_Ca_. Both the dependence of the open probability on the applied voltage and the values of the mean open and closed dwell times correspond to the mitoBK_Ca_ channel from cardiac tissue and are close to the values observed for endothelial and glioma mitoBK_Ca_^7,12,22^.

Apart from that the activity of the channel was dependent on the presence of calcium ions. This property is one of the key features of BK_Ca_ channels^50^. Furthermore, application of the BK_Ca_ activator NS11021 induced an increase in the ion current in the presence of 100 µM calcium ions. NS110121 was previously shown to be a potent activator of BK_Ca_ from the plasma membrane when channels were expressed in oocytes of *Xenopus laevis* and HEK293 cells^38^. It was also shown that activation of cardiac mitoBK_Ca_ after application of NS11021 leads to the induction of cytoprotection mechanisms^51,52^. This compound activates BK_Ca_ channels over a range of calcium ion concentrations including nominally Ca^2+^-free medium and truncated channels lacking calcium-sensing domain^39^. Contrary to the aforementioned studies, in our experiments no increase in channel activity was observed after the administration of NS11021 in the presence of 1 µM Ca^2+^ in experimental buffer. One of the possible explanations for the observed differences could be mitochondrial localization of the channel and potential interactions with the mitochondrial proteome and/or differences in lipid composition between mitochondria and the plasma membrane. On the other hand, it is possible that recordings in the wider range of voltages would confirm the channel activation by NS11021 in the presence of 1 µM Ca^2+^. However, in our system, the use of higher voltages during mitoplast patch-clamp experiment results in membrane instability. Additionally, NS11021 was shown to modulate mitochondrial functions, and these effects were reversed by the application of paxilline, proving the involvement of the mitoBK_Ca_ channel^7^. In our experiments, paxilline inhibited channel activity. Similarly, the application of the mitoBK_Ca_ inhibitor hemin resulted in a decrease in the probability of channel opening. Previously, heme and hemin were shown to block mitoBK_Ca_ activity in glioma and endothelial cells^27–29^. Importantly, both inhibitors block plasma membrane BK_Ca_ channels^41,53^.

All of the above data strongly support the hypothesis that the VEDEC splice variant of the BK_Ca_ channels forms a functional channel in the inner mitochondrial membrane. The expression of VEDEC in HEK293T cells results in the presence of the α subunit in various cellular compartments, including the endoplasmic reticulum and mitochondria. This observation is in line with previously published data^35,37,54^. Equally importantly, in contrast to HEK293T cells in cardiac tissue, the VEDEC splice variant is exclusively targeted to mitochondria^35^. However, the VEDEC isoform is not the only splice variant that could be targeted toward mitochondria. In astrocytoma U87-MG cells, the full-length VEDEC isoform was not detectable, but mitoBK_Ca_ activity was present^55^. Additionally, it was shown that the VYR and ERL isoforms of BK_Ca_ might be located in mitochondria after expression in HEK293 cells^44,54^. These observations might suggest that in cardiac cells, a specific VEDEC sorting mechanism exists.

Additionally, our study shows that HEK293T cells could be a promising experimental model for expression and electrophysiological measurements of mitochondrial potassium channels. HEK293 cells are a common model in studies of BK_Ca_ channels in the plasma membrane. Some previous studies suggested s lack of BK_Ca_ pore-forming subunit expression in these cells^40^. On the other hand, native expression of BK_Ca_ in HEK293 cells was shown recently, and patch-clamp recordings suggested the existence of minimal currents corresponding to BK_Ca_ activity in the plasma membrane^41,44^. In our cells we did not observe any known mitoK channel electrophysiological activity in mitoplasts isolated from wild-type HEK293T cells. Therefore, in our opinion, HEK293T cells could be a suitable model for studies of these issues related to mitoBK_Ca_ in the future.

## Methods

### Cell culture

HEK293T cells were a kind gift from the laboratory of Prof. Mike Ryan (Monash University, Australia) and Dr. David Stroud (currently The University of Melbourne, Australia). The cells were grown in Dulbecco’s modified essential medium (Hirszfeld Institute of Immunology and Experimental Therapy, Wrocław, Poland) supplemented 10% FBS (Gibco), GlutaMAX (Gibco), uridine 50 μg/ml, 100 U/ml penicillin, and 100 µg/ml streptomycin (Sigma-Aldrich) at 37 °C in a humidified atmosphere with 5% CO_2_.

### Cell transfection for electrophysiological studies

Plasmid DNA encoding the VEDEC splice variant (previously described by Singh et al.^36^ was transiently introduced into HEK293T cells by transfection with polyethylenimine (PEI, Sigma-Aldrich). Briefly, the cells were seeded on 150 mm plastic dishes and after reaching 60 – 80% of confluency transfection was performed. First, 20 µg of plasmid DNA was resuspended in 1 ml of Optimem (Invitrogen). In parallel 60 µg of PEI was resuspended in 1 ml Optimem. After 5–15 min of incubation both solutions were mixed and incubated for 30 min. Next, the culture medium was replaced, and a mixture of DNA/PEI was added to the cells. After 3–4 h incubation, the Optimem was replaced with culture medium. After 20–24 h, mitochondria were isolated for patch-clamp experiments.

### Mitochondria isolation

Mitochondria were prepared as previously described^56^. For the patch-clamp experiments, HEK293T cells were washed and collected in PBS buffer and centrifuged for 8 min at 800×*g*. Next, the pellet was resuspended in ice-cold isolation buffer (sucrose 250 mM, HEPES 5 mM, EDTA 1 mM, pH 7.2) and gently homogenized with a Dounce homogenizer. To isolate the mitochondrial fraction, the homogenate was centrifuged for 10 min at 9200×*g* and 4 °C. Next, the pellet was resuspended and centrifuged at 750×*g* for 10 min, and 4 °C. The resulting supernatant was centrifuged in a new tube for 10 min at 9200×*g*, 4 °C. The pelleted mitochondrial fraction was resuspended in an isotonic solution (150 mM KCl, 10 mM HEPES, pH 7.2). The tubes and homogenizer were cooled on ice. Each mitochondrial isolation was performed using between 1 and 3 million cells.

### Electrophysiological single-channel recordings

Patch-clamp experiments using HEK293T cell mitoplasts were performed as previously described^23,57^. In brief, a patch-clamp pipette was filled with an isotonic solution containing 150 mM KCl, 10 mM HEPES, and 100 µM CaCl_2_ at pH 7.2. All of the modulators of the mitoBK_Ca_ channel were added as dilutions in isotonic solution. For the calcium-dependence experiments, the concentration of free Ca^2+^ was controlled with EGTA, and appropriate concentrations of CaCl_2_ were calculated with MaxChelator software^58^ (Stanford University, Stanford, CA, USA). To apply channel modulators and isotonic solutions with different calcium concentrations, a perfusion system was used. The mitoplasts at the tip of the measuring pipette were transferred into the openings of a multibarrel “sewer pipe” system in which their outer faces were rinsed with the test solutions (Fig. 1A)^23^. The current–time traces of the experiments were recorded in single-channel mode. The pipettes were made of borosilicate glass and had a resistance of 10–20 MΩ (Harvard Apparatus GC150-10)^23^. A PC-10 puller (Narishige) was used. The currents were low-pass filtered at 1 kHz and sampled at a frequency of 100 kHz (amplifiers: Axopatch 200B, digidata: Axon 1440A, Molecular Devices)^23^. The traces of the experiments were recorded in single-channel mode. For data analysis, Clampfit 10.7 software (Axon Instruments, Molecular Devices) was used. The conductance of the channel was calculated from the current–voltage relationship (Fig. 2B)^23^. The open probability (P_o_) of the channels was determined using the single-channel search mode. Changes in the ion current were determined by event statistics. For multichannel recordings, the area (pA*ms) under the curve was calculated. The baseline was fixed individually for each experiment based on the closed state of the channels (pA). The maximum number of channels in the patch was obtained by dividing the peak amplitude (pA) by the current of a single channel (pA). Fitting the Boltzmann function to the data from the open probability/voltage plot (Fig. 2C) and the calculation of the V_1/2_ value and the gating charge were prepared according to GraphPad Prism software.

All modulators of the mitoBK_Ca_ channels used in the study were from Sigma-Aldrich.

The electrophysiological data for transiently transfected HEK293T cells were obtained from 56 independent mitochondrial isolations (approx. 600 patches). A mitoplast patch-clamp of untransfected HEK293T cells was acquired from 10 independent mitochondrial isolations (104 patches).

### Reverse-transcription, qualitative and quantitative PCR

RNA from HEK293T cells was isolated using the RNAesy Mini Kit (QIAGEN) according to the manufacturer’s instructions. Reverse-transcription reactions were performed using RevertAid First Strand cDNA Synthesis Kit (Thermo Scientific). For analysis of the expression levels of selected genes, SYBR Select master mix (Applied Biosystems) was used. The reaction was performed with a 7900HT real-time PCR system (Applied Biosystems). For standard PCR, REDTaq polymerase (Sigma-Aldrich) was used, and a Bio-Rad C1000 Thermal Cycler was applied. Primer sequences used in the study were as follows:

BK α (recognizing all isoforms) forward 5′-CCCGCAGACACTGGCCAATAG-3′, reverse 5′-GAGCATCTCTCAGCCGGTAA-3′;

VEDEC, forward 5′-GGGACAAACAGAATGCAACA-3′, reverse 5′-GGTACTCATGGGCTTGATTT-3′;

GAPDH forward 5′-TCAGACACCATGGGGAAGGTGAA-3′, reverse 5′-GAATCATATTGGAACATGTAAACCATG-3′;

MT-CO1 forward 5′-TTAGCTGACTCGCCACACTC-3′, reverse 5′-GGCCACCTACGGTGAAAAGA-3′.

### Western blot analysis

Mitochondrial fractions isolated from HEK293T cells were isolated as described above. First, a 50 µg sample solubilized in Laemmli buffer (Bio-Rad) was separated by 10% Tris-Tricine gel electrophoresis and transferred onto polyvinylidene difluoride (PVDF) membranes (Bio-Rad). After protein transfer, the membranes were exposed to an anti-BK α antibody (NeuroMab, clone L6/60, diluted 1:200) and an anti-mHSP75 antibody (Abcam, 1:1000). The blots were developed using a secondary anti-rabbit or anti-mouse antibody (both GE Healthcare) coupled to horseradish peroxidase in conjunction with an enhanced chemiluminescence solution (GE Healthcare). The results are the summary of analysis of six independent mitochondrial isolations.

### Immunostaining and confocal microscopy

HEK293T cells were seeded onto a 35 mm glass-bottom dish (IBL Baustoff + Labor GmbH) in standard culture medium. After reaching 50–80% of confluency, the culture medium was replaced with DMEM supplemented with 10% FBS and glutamine (as above) but without antibiotics. Next, the cells were transiently transfected with the appropriate plasmid DNA. DNA (2.5 µg VEDEC-encoding plasmid) and 2.5 µl of PEI (1 µg/µl in water) were resuspended in 50 µl of Optimem (Invitrogen). After 15 min of incubation at room temperature, the mixture was added to the cells. After approximately 24 h, the cells were fixed for 15 min with an ice-cold 4% solution of paraformaldehyde (Cell Signaling Technology). For mitochondrial visualization prior to fixation the cells were incubated with 100 nM MitoRed (Sigma-Aldrich) for 30 min at 37 °C in a humidified atmosphere with 5% CO_2_. After fixation, the cells were washed with PBS and permeabilized with 0.05% Triton X-100 solution in PBS (2 × 3 min). Next, the cells were washed with PBS and incubated overnight at 4 °C with a primary antibody resuspended in PBS supplemented with 4% FBS. For indication of the BK_Ca_ α subunit, a 1:200 anti-BK α antibody was used (Alomone Labs, APC021). For indication of the endoplasmic reticulum, a 1:500 anti-PDI (protein disulfide isomerase) antibody was applied (Abcam). Next, the cells were washed with PBS and incubated with a secondary antibody for 1 h at RT (1:1000, anti-mouse Alexa 555, anti-rabbit Alexa 488). Finally, the cells were washed with PBS and mounted with Vectashield medium (Vector Laboratories). Confocal images of stained cells were acquired using an Olympus FV 1200.

### Statistical analysis

The results are presented as means ± SD obtained from at least three independent experiments. Unpaired two-tailed Student’s t-test was used to identify significant differences between two groups of samples. For analysis of differences among at least three groups the one-way ANOVA followed by Tukey’s multiple comparison test was used. Differences were considered to be statistically significant if p < 0.05 (*), p < 0.01 (**), or p < 0.001 (***).

## Supplementary Information

Supplementary Information.

## Abbreviations

P_o_: Open probability of the channel
mitoBK_Ca_: Mitochondrial large conductance calcium-activated potassium channel
mitoK: Mitochondrial potassium channels
KCOs: Potassium channel openers
BK_Ca_: Large conductance calcium-activated potassium channel
