# Supplementary material for: Single channel properties of mitochondrial large conductance potassium channel formed by BK-VEDEC splice variant
Source: Sci Rep. 2021 May 25;11:10925. doi: 10.1038/s41598-021-90465-3 (PMC8149700; doi:10.1038/s41598-021-90465-3)
Supplement: Supplementary file 1 — Supplementary Information. [file 41598_2021_90465_MOESM1_ESM.pdf]

Single channel properties of mitochondrial large conductance potassium channel formed by BK-VEDEC splice variant

Shur Gałecka[1*], Bogusz Kulawiak[1$*], Piotr Bednarczyk[2], Harpreet Singh[3], Adam Szewczyk[1]

[1]Laboratory of Intracellular Ion Channels, Nencki Institute of Experimental Biology, Polish Academy of Sciences, Pasteura 3, 02-093 Warsaw, Poland;

[2]Department of Physics and Biophysics, Institute of Biology, Warsaw University of Life Sciences-SGGW, Nowoursynowska 166, 02-787 Warsaw, Poland;

[3]Department of Physiology and Cell Biology, The Ohio State University, Columbus, OH, 43210, USA.

*both authors contributed equally

[$]To whom correspondence should be addressed. Bogusz Kulawiak: Laboratory of Intracellular Ion Channels, Nencki Institute of Experimental Biology, Polish Academy of Sciences, 3 Pasteur St., 02-093 Warsaw, Poland; b.kulawiak@nencki.edu.pl; Tel. (48) 22 58 92 343

Figure S1

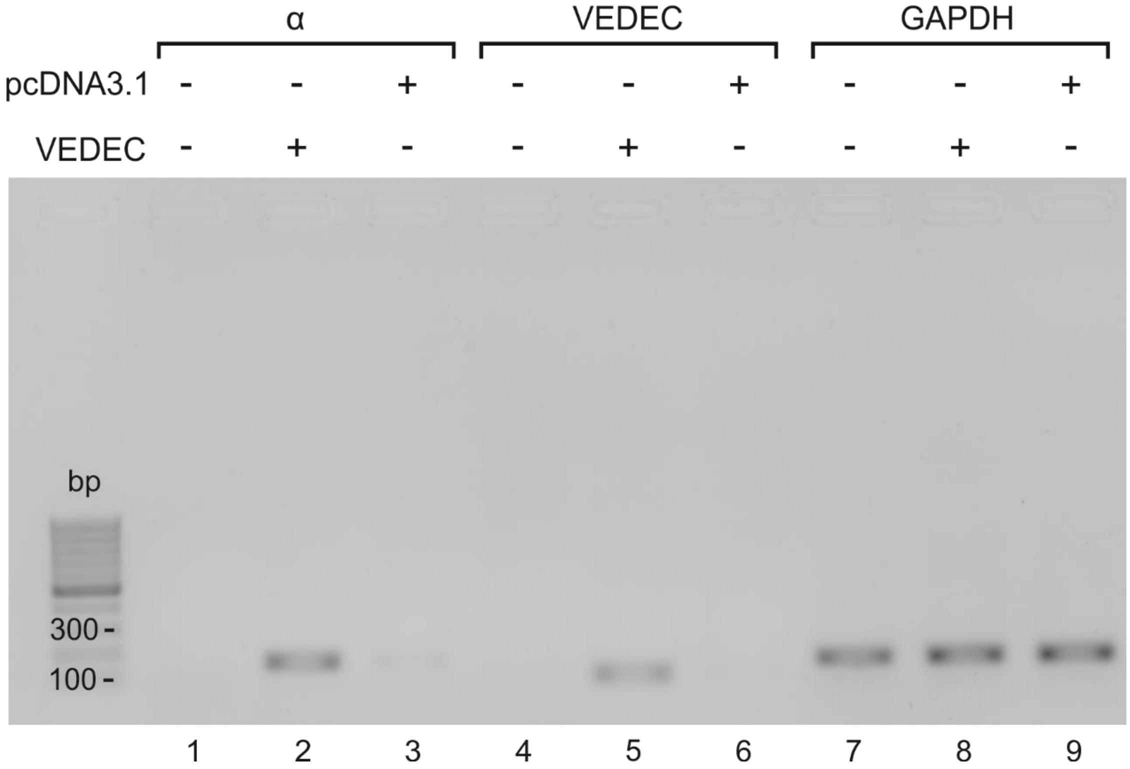

Figure S1. Analysis of selected gene expression using qualitative PCR in wild-type and VEDEC transiently transfected HEK293T cells. Full length of the agarose gel presented in the Figure 6A.

Figure S2

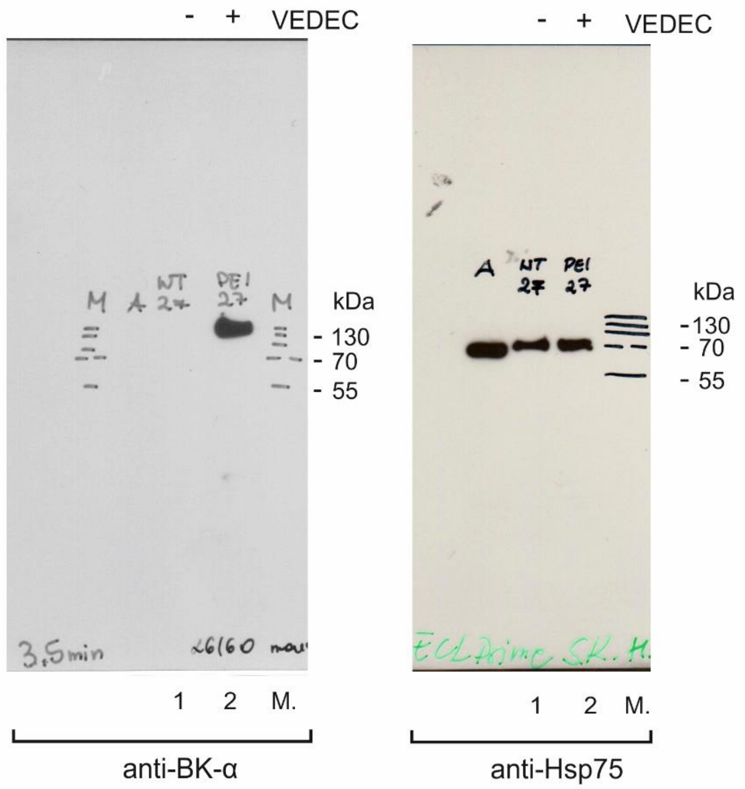

Figure S2. Western blot analysis of the BK_{Ca} pore-forming subunit in the mitochondrial fraction of wild-type and VEDEC transiently transfected HEK293T cells. Full length of the PVDF membranes presented in the Figure 6C. The membrane was cut below 55 kDa marker and then was initially probed with an anti-BKα antibody (NeuroMabs, clone L6/60, diluted 1:200). Afterwards it was reprobed with an anti-mHSP75 antibody (Abcam, 1:1000). The blots were developed using a secondary anti-rabbit or anti-mouse antibody (both GE Healthcare) coupled to horseradish peroxidase in conjunction with an enhanced chemiluminescence solution (GE Healthcare).
